# Exposure to Toxic Compounds Using Alternative Smoking Products: Analysis of Empirical Data

**DOI:** 10.3390/ijerph22071010

**Published:** 2025-06-26

**Authors:** Sandra Sakalauskaite, Linas Zdanavicius, Jekaterina Šteinmiller, Natalja Istomina

**Affiliations:** 1 Laboratory of Immunology, Department of Immunology and Allergology, Lithuanian University of Health Sciences, 50161 Kaunas, Lithuania; 2 Faculty of Medicine, Vilnius University, 03101 Vilnius, Lithuania; 3 Nursing Department, Tallinn Health Care College, 13418 Tallinn, Estonia; 4 Faculty of Public Governance and Business, Mykolas Romeris University, 08303 Vilnius, Lithuania

**Keywords:** carcinogens, carbonyls, emissions, ENDS, harmful and potentially harmful constituents, heat not burn tobacco, harm reduction, PAHs, vaping, VOCs

## Abstract

Tobacco control policies have aimed to reduce the global prevalence of smoking. Unfortunately, the recent survey data shows that about 24% of Europeans still smoke. Although combustible cigarettes remain the most used tobacco product, the tendency made evident in the prevalence of smoking-alternative nicotine-containing products increases. Studies that can objectively assess the long-term health effects of the latter products are lacking, so assessing toxic substances associated with smoking-alternative products and comparing them to substances from combustible cigarettes could inform future public health efforts. The manufacturers of these alternative products claim that the use of alternatives to combustible cigarettes reduces exposure to toxic compounds, but the reality is unclear. This study compares the concentrations of toxic substances in generated aerosols and performs calculations based on mainstream cigarette smoke and aerosols from smoking-alternative products. It summarizes the amounts of harmful and potentially harmful constituents per single puff. Alternative smoking products are undoubtedly harmful to non-smokers. Still, based on the analysis of the latest independent studies’ empirical data, the concentrations of inhaled HPHCs using heated tobacco products or e-cigarettes are reduced up to 91–98%, respectively; therefore, for those who cannot quit, these could provide a less harmful alternative. However, more well-designed studies of alternative product emissions are needed, including an analysis of the compounds that are not present in conventional tobacco products (e.g., thermal degradation products of propylene glycol, glycerol, or flavorings) to evaluate possible future health effects objectively.

## 1. Introduction

Globally, according to the surveys of the World Health Organization, the prevalence of smoking was about 22% in 2020 [1]. An assessment of smoking trends between 1970 and 2020 shows that smoking rates declined by about 15% within the US and several other countries in the Organization for Economic Cooperation and Development (OECD) [2]. Tobacco control policies over the past two decades have aimed to reduce the global prevalence of smoking, but the recent special Eurobarometer 539 survey of 2023 shows that about 24% of people in the EU still smoke. This is only 1% less than in 2021 [3]. Furthermore, according to the World Health Organization (WHO), the tobacco epidemic is one of the world’s biggest public health threats, killing more than 8 million people worldwide every year [4,5]. Tobacco use is associated with the risk of developing a number of diseases: cardiovascular, respiratory and oncological diseases; reproductive health problems; and adverse effects on the immune system [4,6,7]. Smoking cessation is difficult because smoking addiction is caused by physiological (nicotine) and psychological factors (smoking ritual) [8,9]. For these reasons, the search has begun for ways in which smoking behaviour can still be changed to minimise the damage to health. This has led to the development of new products that manufacturers claim are less harmful alternatives to combustible cigarettes (CC). These include non-combustible tobacco products (e.g., heated tobacco products (HTPs), pin heating systems) and nicotine products (e.g., e-cigarettes, nicotine pads) [10,11]. The problem is that alternative products became popular long before there was sufficient scientific evidence to determine their potentially harmful health effects on consumers [12]. Exposure to toxic compounds found in tobacco smoke has been consistently linked to an increased risk of various diseases. While it is widely assumed that reducing exposure to harmful and potentially harmful constituents (HPHCs) would lower disease risk, direct evidence establishing a causal link between reduced exposure and decreased disease incidence remains limited. To date, few studies have systematically evaluated how the use of non-combustible tobacco products influences toxicant exposure levels, and these studies are tobacco industry-dependent [13,14,15,16]. Unfortunately, there is no data about the relationship between exposure to toxicants and disease risk. Nevertheless, the first step is to clarify the level of reduction in toxicant emissions associated with non-combustible tobacco products, based on independent studies. Therefore, our study aims to summarize and compare the emission levels of HPHCs in non-combustible tobacco products, providing a basis for further research studies on the long-term health effects of these products.

### 1.1. The Combustion Process of the Cigarette

Combustion-based products are the ones most harmful to smokers. Cigarette combustion produces ash and smoke containing particulate matter and large quantities of more than 7000 identified harmful chemicals, of which almost 100 are classified as harmful or potentially harmful because of their association with smoking-related diseases [17,18]. One group of pollutants with carcinogenic and mutagenic properties comprises polycyclic aromatic hydrocarbons (PAHs), which have a temperature-dependent profile: PAH formation starts at temperatures up to 450 °C, with a maximum yield at 500–550 °C. Therefore, if the process is carried out at temperatures below 400 °C, the release of toxic substances is significantly reduced [19,20]. This is the rationale behind the principle of HTPs as an alternative to combustible cigarettes. In non-combustible tobacco products, electronically controlled heating prevents combustion. Natural tobacco containing nicotine is heated and no ash is produced [21,22]. As a result, the smoker inhales an aerosol of heated tobacco. Thus, compared to cigarettes, harmful substances should be formed in lesser quantities, but what is the reality? There is a lack of studies comparing all three products: combustible cigarettes, HTP, and e-cigarettes. Furthermore, it is not easy to compare the levels of inhaled HTP compounds from separate studies because the study conditions need to be taken into account. For this reason, in the present work, we have performed calculations to estimate the levels of inhaled harmful and potentially harmful constituents (HPHCs)-based compounds in different smoking products and compared them with the levels associated with smoke inhaled while cigarette smoking.

### 1.2. Comparison of the Principles of Operation of HTP and E-Cigarettes

One common problem is that users equate HTPs with e-cigarettes. However, these two products have different principles of operation. In HTPs, the natural tobacco containing nicotine is heated [23]. In contrast, smoking e-cigarettes vaporizes the e-cigarette liquid. This liquid consists of vegetable glycerol (60–70%), propylene glycol (25–30%), nicotine extracted from tobacco (0–6%), and flavorings (5–15%). The glycerol and propylene glycol in e-liquids cause smokers to exhale a large amount of vapor, which is commonly referred to, by users, as smoke. As with HTPs, no ash is produced [24,25,26]. Thus, the main difference between HTPs and e-cigarettes is that the latter uses a vaporized chemical liquid, and the smoker inhales the vapor. In contrast, users of HTPs inhale an aerosol of heated tobacco [23,27,28].

### 1.3. Comparison of Emissions

The Food and Drug Administration (FDA) of the United States of America has published a list of “Harmful and Potentially Harmful Constituents (HPHCs) in Tobacco Products and Tobacco Smoke”; there are five main groups of compounds in this list: carbonyls, volatile organic compounds, N’-nitrosamines, heterocyclic compounds, and inorganic compounds (metals) [27]. The World Health Organization (WHO) has also published a list of chemicals which are recommended as candidates for mandatory lowering of levels [28,29]. Therefore, in this paper, we will compare the emissions of the most hazardous, high-priority substances present in combustible cigarette smoke and the mainstream aerosol smoking-alternative products and will summarize the data from the latest independent studies.

## 2. Methods

This research consisted of three steps. First, based on a review of the literature, we collected the emission results for HPHCs in mainstream CC smoke and alternative product aerosols, based on data from independent studies. Then, we converted the concentrations of HPHCs into the concentrations obtained per single puff. Finally, we calculated the reduction in HPHCs emissions using Formulas (1) and (2).

### 2.1. Criteria for Literature Selection

A comprehensive search was performed across two electronic databases, PubMed and Science Direct, covering publications from January 2014 to May 2025. The search strategy included keywords such as “toxic compounds in cigarette smoke” (result: 456 publications), “toxic compounds in heated tobacco product aerosols” (result: 37 publications), “toxic compounds in e-cigs aerosols” (result: 100 publications), “comparison of emissions from smoking products” (result: 105 publications), and their respective synonyms. To facilitate comparisons of the results, we have chosen only studies that are not associated with the tobacco industry. We have selected studies that employed validated methods, including those conducted under the Health Canada Intense (HCI) and International Organization for Standardization (ISO) regimens. Given that e-cigarette emissions depend on the flavor added, we used the results obtained with tobacco-flavored e-cigarettes and HTP regular products. Fifteen studies met the inclusion criteria and were included for further analysis.

### 2.2. Data Normalization/Conversion

To date, we were unable to identify any independent studies that simultaneously examined all three smoking products—CC, HTPs, and e-cigarettes. Therefore, the substance concentrations measured by a whole-product unit (CC, HTPs, or e-cigarettes) reported in the reviewed publications were converted, using the available data, to reflect the estimated amount per inhalation (i.e., concentration per puff), enabling standardized comparisons across studies. A reference cigarette, 3R4F, was chosen to objectify the emissions from combustible cigarettes (as a comparator) [30].

### 2.3. Statistical Analysis Methods

Descriptive statistics, including means, medians, and minimum and maximum values, were calculated using Microsoft Excel 2010 (Microsoft Corporation, Redmond, WA, USA).

#### The Calculation of the Risk Reduction Potential of HPHCs

The average concentrations per single puff according to the data from different independent studies were calculated. To assess the differences between the levels of toxic compounds in the emissions of the different smoking products, calculations according to Formula (1) below have been applied in the calculation of the reduction:

(1) $$\mathrm{Reduction}\;=\;(1\;-\;\frac{Average\;concentration\;of\;toxic\;compound\;in\;HTP\;or\;E-cig}{Average\;concentration\;of\;toxic\;compound\;in\;CC})\;\mathrm{\times 100\%}\;$$

For the deriving the average reduction for all toxic compounds, we applied Formula (2) below:

(2) $$\mathrm{Average}\;\mathrm{reduction}\;=\;\frac{Sum\;of\;the\;percentage\;reductions\;for\;each\;toxic\;compound}{Number\;of\;toxic\;compounds\;assessed}$$

The formulas applied in our study are based on standard comparative-assessment logic and are commonly used in exposure reduction evaluations. This approach provides a general indicator of overall emission reduction across a range of analytes when toxicological weighting is not applied.

## 3. Results

### 3.1. Comparative Data on Smoking Product Emissions

Based on empirical data from the meta-analysis of scientific research data and scientific articles, median and min-max ranges of the substances’ concentrations per single puff were calculated from the collected data and are shown in Table 1.

### 3.2. Reduced Emission Profiles of Alternative Products

**Figure 1:**
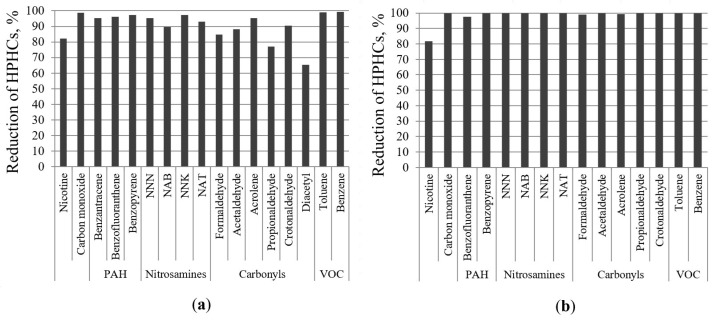
The reduction (%) in HPHCs between combustible cigarettes and alternative products: (**a**) heated tobacco products (HTPs) and (**b**) e-cigarettes (E-cigs). The percentage of reductions were calculated in comparison with 3R4F.

The results of the reduction calculations are presented in Figure 1. Data analysis showed that the inhaled levels of HPHCs that are released during the combustion process, such as polycyclic aromatic hydrocarbons (PAHs) and volatile organic compounds (VOCs), are reduced 99% and 100% in e-cigarettes and 96% and 99% in HTPs aerosols. The concentrations of inhaled carbonyls are reduced by almost 100% in e-cigarettes and 65–95% in HTPs aerosols. The inhaled concentrations of the most important group of carcinogens in tobacco products—tobacco-specific nitrosamines (TSNAs)—are reduced 99% in e-cigarettes and 89–94% in HTP aerosols. The nicotine and CO concentrations per single puff are reduced by 81% and 99% in e-cigarettes and 82% and 98% in HTPs aerosols, respectively. Some substances in the emissions of the alternative products were under the detection limit (e.g., NAB, NNK, propionaldehyde, crotonaldehyde, VOC, and some metals). Therefore, these reduction rates could not be evaluated. However, such a result showed that the amounts of these substances in the aerosols of mainstream HTPs or E-cigs is reduced compared with CC.

## 4. Discussion

A smoking reduction strategy aims to reduce the number of smokers, but this is not yet an easy task because quitting smoking is a long-term process, and the risks to smokers’ health need to be reduced as soon as possible. Our analysis of independent studies showed that the concentrations of HPHCs indicated in the FDA and WHO lists are decreased by up to 91% in HTP aerosols and 98% in e-cigarettes, per puff. Such a significant reduction in HPHCs results in a reduced exposure to toxic substances, which in turn leads to fewer adverse health effects for smokers. Still, it is important to note that alternatives to combustible cigarettes are not harmless, and they are targeted to smokers who are unable to quit.

A correctly modeled study design and identical experimental conditions are essential in order to assess the emissions from different tobacco products objectively. However, the analysis of the current data revealed the problem that many studies are inadequately designed, and cannot objectively assess whether the alternatives to combustible cigarettes are less harmful to human health. It was found that the results for the emissions of the reference cigarette were not the same in different studies. This is because the researchers use different methodologies and equipment, which are not necessarily validated, in their studies. In addition, some analytes, such as CO emissions, are associated in e-cigarettes with higher power settings, longer puff durations, and e-liquids with flavors [72]. Thus, when comparing studies, it is important to consider that the differences between results obtained could be due to methodological aspects. Therefore, the emissions of smoking-alternative products should be critically evaluated. It should be noted that there are only two validated regimens of analytical methods for toxic compound determination in smoke: ISO and CI [53]. Thus, evaluations of the alternatives to combustible cigarettes should be based only on studies that use reliable equipment and validated analysis protocols. Furthermore, while evaluating and summarizing the effects of alternative products on human health, the most common errors are small sample sizes and inappropriate comparisons between study groups (comparing non-smokers with smokers). Since there is no doubt that these products have an adverse effect on human health, non-smokers should not be included in the subject groups for the purposes of comparative analysis.

Interestingly, two substances to which not much attention is given are the key components of e-cigarettes, glycerol and propylene glycol, which are not included in the list of “Harmful and Potentially Harmful Constituents (HPHCs) in Tobacco Products and Smoke.” The food industry has long used both compounds as sweeteners or stabilizing agents, and they are considered safe to ingest. However, the consequences of propylene glycol and glycerol inhalation remain unknown. There is data that these compounds are associated with increased upper-airway symptoms and could impact the response to concomitant exposure to pollutants, allergens, and pathogens [73,74,75]. Additionally, the health effects of the thermal degradation products from the flavorings used in E-cigs remain poorly understood. Many flavorings can break down into new compounds during heating, some of which, such as aldehydes or reactive carbonyls, may pose a risk to respiratory health. However, the current scientific evidence on the inhaled amounts and long-term effects of these substances is limited [76,77]. Therefore, these results provide a basis for a deeper examination of the long-term effects of these compounds on human health. It is also important to note that, in parallel to regulated and commercially available products, the spread of non-regulated E-cigs products, which often contain undeclared components, remains a major public health concern. These products are more likely to be accessed by young people and have been implicated in various acute health incidents, underscoring the need for strict market surveillance and regulation.

The limitation affecting our publication is that the data was collected from separate studies, the results of which may have been influenced by technical and methodological differences (e.g., differences in protocols or detection limits). Nevertheless, we have analyzed a large amount of empirical data based on the analysis of generated aerosol and carried out calculations that allowed us to assess the concentrations of HPHCs in smoking products as objectively as possible. This suggests the necessity for studies that simultaneously analyze emissions from all three smoking products. In this study, we focused on comparing only those HPHCs that the WHO and the FDA specifically recommend reducing in tobacco products. These include compounds commonly found in both traditional cigarettes and alternative products. However, we did not include substances that are unique to E-cigs—such as flavoring agents, solvents like propylene glycol and glycerol, or other potentially harmful VOCs. However, our study contributes to evidence-based rather than emotion-based claims about the concept of harm reduction through the use of alternative tobacco products. The ultimate goal is to reduce the damage smoking causes to the health of smokers who cannot quit [9,10] and a reduction in the emissions of HPHCs is the first step towards reduced harm.

## 5. Conclusions

Based on our analysis of data from independent studies, both heated tobacco product aerosols and e-cigarette vapor contain significantly lower levels of toxicants—specifically those identified for reduction by the FDA and WHO—compared to combustible cigarette smoke, by approximately 91% and 98%, respectively. Although alternative products are not free of harmful and potentially harmful constituents, the observed reduction in emission levels suggests a substantially lower exposure to toxic substances for subjects who cannot quit. It is generally accepted that reduced exposure to toxicants is likely to correspond to reduced health risks. Therefore, our findings provide a basis for future research into the potential health effects of switching from traditional cigarettes to non-combustible alternatives. In any case, adequately designed studies are needed to assess the long-term effects of these products.

## Figures and Tables

**Table 1:** Table summarizing the median concentrations of the determined HPHCs per single puff.

Compound	Harm Caused	Concentration per 1 Puff Median [Min–Max]			References
		3R4F	Heated Tobacco Products	Electronic Cigarettes	
Nicotine, mg	Causes addiction, has inflammatory and anti-inflammatory properties [31].	0.17 [0.09–0.22]	0.1 [0.09–0.11]	0.05 [0.02–0.32]	[32,33,34,35,36]
Carbon monoxide, mg	Is associated with chronic carboxyhaemoglobinemia and the development of cardiorespiratory disease [37].	2.78 [1.32–3.7]	0.04 [0.03–0.06]	0.002 [0.001–0.003]	[33,35,36,38,39,40]
Polycyclic aromatic Hydrocarbons (PAH)					
Benz[a]anthracene	Oxidative stress inducer leading neuronal damage [41].	2.84 [2.47–3.21]	0.13 [0.12–0.22]	<LOD	[38,40,42,43]
Benzo[b+k]fluoranthene, ng	Carcinogen [44].	0.62 [0.4–0.83]	0.03 [0.01–0.04]	0.016 [0.01–0.018]	[38,40,42,43]
Benzo[a]pyrene, ng	The most potent carcinogen among polycyclic aromatic hydrocarbons [45].	1.40 [0.69–1.67]	0.05 [0.04–0.06]	0.003 [0.002–0.006]	[38,40,42,43]
Tobacco-specific Nitrosamines					
N′-nitrosonornicotine (NNN), ng	Carcinogen [46].	24.68 [12.89–34.57]	0.96 [0.87–1.75]	- [<LOD–0.002]	[32,33,35,36,38,47]
N′-nitrosoanabasine (NAB), ng	Carcinogen [46].	2.67 [1.6–3.64]	0.26 [0.2–0.47]	- <LOD	[32,33,35,36,38,47]
4-(Methylnitrosamino)-1-(3-pyridyl)-1-butanone (NNK), ng	Carcinogen [46].	22.25 [14.3–27.82]	0.61 [0.2–0.88]	- [<LOD–0.004]	[32,33,35,36,38,47]
N′-nitrosoanatabine (NAT), ng	Carcinogen [46].	20.98 [14.3–27.82]	1.51 [1.23–1.75]	- [<LOD–0.005]	[32,33,35,36,38,47]
Carbonyls					
Formaldehyde, μg	Carcinogen [48], has a toxic effect at the cellular level. Causes irritation of the airways and damage to airway cells, a source of contact dermatitis [49].	4.7 [1.88–8.09]	0.53 [0.11–1.9]	0.04 [0.03–0.12]	[32,34,35,36,43,47,50]
Acetaldehyde, μg	One of the most common carcinogens in cigarette smoke [51].	127.1 [136.4–154]	16.5 [9.5–18.26]	0.01 [0.01–0.1]	[32,34,35,36,43,47,50]
Acroleine, μg	Promotes endothelial dysfunction, oxidative stress, dyslipidemia, and platelet activation [52]. Chronic exposure to acrolein through cigarette smoke has been associated with asthma, acute lung damage, chronic obstructive pulmonary disease (COPD), and respiratory cancer [53,54,55,56].	12.37 [11.82–16.22]	0.67 [0.04–0.94]	0.06 [0.003–0.17]	[32,34,35,36,43,47,50]
Propionaldehyde, μg	Causes cough and sore throat [57].	10.67 [6.2–13.02]	1.07 [0.56–1.61]	- [<LOD–0.002]	[32,34,35,36,43,47,50]
Crotonaldehyde, μg	A potent eye, respiratory, and skin irritant, associated with cardiopulmonary toxicity and cardiovascular disease [56,58,59].	4.36 [1.29–4.7]	0.4 [0.05–0.83]	- [<LOD–0.001]	[35,36,47,60,61]
Volatile organic compounds					
Toluene μg	Negatively affects the brain and central nervous system [62]	14.89 [10.38–21.41]	0.14 [0.12–0.22]	- [<LOD–0.003]	[34,35,36,38,43]
Benzene, μg	Increases the risk of leukemia, lymphoma, and cardiovascular disease. Causes a deficiency of circulating angiogenic cells and increases low-density lipoprotein levels [63,64,65].	7.91 [6.62–10]	0.05 [0.04–0.08]	- [<LOD–0.0008]	[34,35,36,38,43]
Inorganic compounds					
Nickel, ng	Genotoxic effects, may increase the risk of oral cancer [66].	0.41 [0.39–1.43]	<LOD	0.0015 [0.001–0.002]	[35,36,47,61,67]
Cadmium, ng	Acute inhalation exposure may result in flu-like symptoms and may damage the lungs. Chronic exposure can result in kidney, bone, and lung disease [68].	11.87 [8.54–15.2]	- <LOD	- <LOD	[35,36,47,61,67]
Chromium, ng	Inhalation may cause respiratory irritation [69].	0.09 [0.09–0.19]	<LOD	<LOD	[35,36,47,61,67]
Lead, ng	Causes oxidative stress in cells, may cause lung cancer, has a strong negative effect on the brain, nervous system and red blood cells [70].	3.26 [2.9–3.62]	- [<LOD–0.76]	- [<LOD-003]	[35,36,47,61,67]
Mercury, ng	May have toxic effects on the nervous, digestive, and immune systems, and on lungs, kidneys, skin, and eyes [71].	0.42 [0.25–0.48]	<LOD	<LOD	[35,36,47,61,67]

LOD—below limit of detection.

## Data Availability

The original contributions presented in this study are included in the article. Further inquiries can be directed to the corresponding author.

## Abbreviations

The following abbreviations are used in this manuscript:

CC	Combustible cigarettes
E-cigs	Electronic cigarettes
HPHCs	Harmful and potentially harmful constituents
HTPs	Heated tobacco products
VOCs	Volatile organic compounds
